# Goblet cell carcinoid of the appendix

**DOI:** 10.1186/1477-7819-3-36

**Published:** 2005-06-20

**Authors:** Payam S Pahlavan, Rani Kanthan

**Affiliations:** 1 Department of Physiology and Pathophysiology, University of Heidelberg, Heidelberg, Germany; 2 Department of Pathology, University of Saskatchewan, Saskatoon, Canada

## Abstract

**Background:**

Goblet cell carcinoid (GCC) of the appendix is a rare neoplasm that share histological features of both adenocarcinoma and carcinoid tumor. While its malignant potential remains unclear, GCC's are more aggressive than conventional carcinoid. The clinical presentations of this neoplasm are also varied. This review summarizes the published literature on GCC of the appendix. The focus is on its diagnosis, histopathological aspects, clinical manifestations, and management.

**Methods:**

Published studies in the English language between 1966 to 2004 were identified through Medline keyword search utilizing terms "goblet cell carcinoid," "adenocarcinoid", "mucinous carcinoid" and "crypt cell carcinoma" of the appendix.

**Results:**

Based on the review of 57 published papers encompassing nearly 600 diagnosed patients, the mean age of presentation for GCC of the appendix was 58.89 years with equal representation in both males and females. Accurate diagnosis of this neoplasm requires astute observations within an acutely inflamed appendix as this neoplasm has a prominent pattern of submucosal growth and usually lacks the formation of a well-defined tumor mass. The mesoappendix was involved in 21.64% followed by perineural involvement in 2.06%. The most common clinical presentations in order of frequency were acute appendicitis in 22.5%; asymptomatic in 5.4%; non-localized abdominal pain in 5.15% and an appendicular mass in 3.09%. The most common surgical treatment of choice was appendectomy with right hemicolectomy in 34.70% followed by simple appendectomy in 24.57%. Concomitant distant metastasis at diagnosis was present in 11.16% of patients with the ovaries being the most common site in 3.60% followed by disseminated abdominal carcinomatosis in 1.03%. Local lymph node involvement was seen in 8.76% of patients at the time of diagnosis. The reported 5-year survival ranges from 60 % to 84%. GCC's of the appendix remains a neoplasm of unpredictable biological behavior and thus warrants lifelong surveillance for recurrence of the disease upon diagnosis and successful surgical extirpation.

**Conclusion:**

GCC of the appendix is a rare neoplasm. Due to its wide range of presentation, this tumor should be considered as a possible diagnosis in many varied situations leading to abdominal surgery. Histopathological features such as increased number of Paneth cells, increased amount of mucin secretion and presence of pancreatic polypeptide may predict a more aggressive behavior. The advocated plan of management recommended for patients with tumors that involve the adjacent caecum or with high-grade tumors with histological features such as an increased mitotic rate involve initial appendectomy with completion right hemicolectomy due to the high possibility of local recurrence with intraperitoneal seeding prior to lymph node involvement and a 20% risk of metastatic behavior. In female patients with GCC of the appendix regardless of age, bilateral salpingo-oophorectomy is advocated. In cases with obvious spread of the disease chemotherapy, mostly with 5-FU and leucovorin is advised. Cytoreductive surgery with adjuvant intraperitoneal chemotherapy can offer improved survival in cases with advanced peritoneal dissemination.

## Background

Cancer of the appendix is an uncommon disease [1]. Appendiceal carcinoids however are found in 1 out of every 300 appendectomies. Since more than 30 years ago, a new variant type of epithelial tumor of appendix has been recognized [2,3]. This tumor is reported under different names including goblet cell carcinoid (GCC), adenocarcinoid, mucinous carcinoid, intermediate type of carcinoid, crypt cell carcinoma, amphicrine (endo-exocrine) neoplasia, composite tumor and microglandular carcinoma [2,4,5]. All names except GCC have been omitted from the current World Health Organization (WHO) classification [6]. In general, GCC (International Classification of Disease for Oncology, ICD-O 8243/3) is a rare neuroendocrine tumor of the vermiform appendix, comprising approximately 6% of appendiceal carcinoids and usually occurs in the pure form [7-9]. Some characteristics including its intramural position in the appendix, the continuity with the basiglandular cells of the mucus membrane, and it's generally well-differentiated suggesting its relation to carcinoid [10]. Nevertheless, some authors say that this tumor is far more closely related to intestinal crypts than to carcinoids and insist on not considering this tumor as a variant of carcinoids [11]. GCC has both endocrine and glandular differentiation [9]. This tumor appears to combine features of epithelial and carcinoid neoplasms and in addition the surface mucosal epithelium is not neoplastic [12]. Furthermore, in comparison to the other carcinoid tumors, GCC is usually larger in size [12].

Subbuswamy *et al *described the first report of GCC in 1974 [10]. This type of neoplasm predominantly occurs in the appendix as a distinct pathological entity [13,14] and has uncertain distinct biological behavior [15]. The histology and biology appears to be intermediate between carcinoid tumors and adenocarcinomas [16]. Some authors believe that this tumor usually presents as a low-grade malignancy and metastases are uncommon [17]. Yet, in general, it is believed that it has a more aggressive natural history than classic appendiceal carcinoids with variable malignant potential; thus, it requires a different surgical approach than is usually advocated for classic carcinoids [3]. The tumor most commonly infiltrates the entire appendix transmurally [6]. Anatomically, GCC's are usually located near the tip of the appendix [18]. Preoperative diagnosis is rare and patients usually present with acute appendicitis [2]. The GCC cells are probably APUD (amine precursor uptake and decarboxylation) derived cells with both mucinous and entrochromafin granules [19]. The number of patients who were diagnosed with this neoplasm based on the Medline search is less than 600 worldwide. The purpose of this study is to review the literature regarding this rare neoplasm to identify diagnostic, clinical, histological features, treatment guidelines and understand the prognosis that is somehow different from the usual appendiceal carcinoid.

### History of GCC of the appendix

Appendiceal tumors that share the histological characteristics of carcinoids and adenocarcinomas have been recognized since the 1960s, with early reports by Gibbs, Bates and Butler, Rosai and Rodriguez [5]. In 1969, Gagne *et al *described 3 unusual tumors of appendix with common features of 1) association of nests of entrochromafin cells with mucus secreting glandular structures; 2) integrity of the appendiceal mucosa, and 3) an infiltrative pattern similar to carcinoid tumors but with the propensity to invade nerves [17]. In 1974, Klein reported three cases of mucinous carcinoid tumor of appendix [20] but the first reported cases under the name of GCC were by Subbuswamy *et al *in 1974 [10]. After that Abt *et al *reported microscopical demonstration of the cells of the other known cases with GCC of the appendix [21]. DiPaola *et al *reported the first case of appendico-vesical fistula due to appendix abscess with GCC in 1976 [22]. In 1978 Warkel *et al *reported 39 cases of GCC and revealed more aspects about this neoplasm [23]. In 1979, Warner *et al *described the origin of these cells as being endodermal in origin and explained that these cells arise from crypt base stem cells [19]. In 1980, Ratzenhofer *et al *explained this neoplasm as amphicrine (endo-exocrine) neoplasia [4]. Recent reports are more focused on the genetic aspects and molecular levels of the pathogenesis of this neoplasm [16,7,24]. Due to the paucity of reported cases and non-specific presentations e.g., acute appendicitis, diagnosis of this neoplasm requires a high degree of astute observations in an otherwise inflamed appendix. This is primarily attributed to its predominant submucosal growth and lack of forming a well-defined mass within the appendix.

### Epidemiologic characteristics and etiopathogenetic factors

GCC accounts for less than 5% of primary tumors of the appendix [25]. Based on a review on 227 patients with GCC of the appendix, GCC occurs equally in men and women and with higher occurrence in the white race [1]. Aizawa *et al *explained that the mean age for GCC is 58.8 years, whereas that for carcinoid tumor is 35.9 years [26]. The mean age at diagnosis of GCC is between malignant carcinoid (38 years) and mucinous adenocarcinoma (60 years) [1]. The mean age for those presenting with more localized tumor is significantly lower, compared to the mean age of those with spread beyond the appendix [27]. In addition, the mean age of goblet cell type is significantly higher than the tubular carcinoid of the appendix [28]. In contrast to appendiceal carcinoids that metastasize in 2 to 5% of cases, GCCs metastasize in 15 to 30% of cases [25].

GCCs of the appendix are amphicrine tumors arising from a pluripotent cell with divergent neuroendocrine and mucinous differentiation and with dysregulation of the cell cycle [15]. There is a large reserve G0/G1 pool of pluripotent cells that remain noninvasive until further genetic instability is acquired by the cell [15]. Some studies demonstrated that p53 mutations play a major role in pathogenesis of some GCCs and most of these mutations are G:C to A:T transitions [16]. However, other studies failed to demonstrate such alterations [8]. Allelic loss of chromosomes 11q, 16q, and 18q is frequent in GCC and ileal carcinoids and maybe has a role in tumorigenesis [8]. There were also three reported cases of GCC of the appendix with concomitant appendiceal epithelial neoplasm. Therefore it is assumed that their maybe a common etiologic factor for both GCC and epithelial neoplasm in these cases with two components and this may be attributed to a divergent differentiation from a single stem cell [12]. If this tumor were a true member of the carcinoid family, then its co-existence with mucinous cysadenoma in two reported rare cases would support the unitary stem cell hypothesis of its origin. If this tumor is a carcinoma of crypt cell origin rather than a carcinoid, then combination with cystadenocarcinoma is an example of adenoma-carcinoma sequence that is extremely rare [29].

In conclusion, based on the review of all published papers for nearly 600 diagnosed patients, the mean age for diagnosing GCC was 58.89 years with equal representation in both genders with male to female ratio of 1:0.84. GCC of the appendix occurs later in life in comparison to conventional carcinoid. The etiopathogenic factor that most authors agree is the occurrence of p53 mutations and this is confirmed in most of the reported analyzed cases.

### Histopathological aspects

Although GCC was previously considered a variant of carcinoid tumor, current evidence suggests that GCC is a distinct tumor with a histogenesis different from that of typical carcinoid [8]. Appendiceal neoplasms that exhibit aggressive behavior contain the typical nests of goblet cells that are the essential elements for inclusion in the category of GCC [23]. As immunohistochemical studies have shown that GCC cells contain lysozyme, secretory component (SC) and immunoglobulin A, it suggests that these tumors are derived from cells similar to the lysozyme-producing cells of the small intestinal crypts [27]. The GCC cells are rather larger than normal goblet cells [10] and arise from a pluripotent cell (stem cell) as intestinal APUD cell with endodermal origin with divergent neuroendocrine and mucinous differentiation [3].

GCCs represent a composite biphasic neoplasm of two morphologically differentiated cells, each with a common endodermal embryogenic origin [2]. In early reports, the GCCs were subdivided into two types: the goblet cell type and the tubular type [23]. Goblet cell type is more common than tubular type [30]. Goblet cell type is composed of smooth-bordered nests, usually 4 or more cells. The tubular type is composed of small, discrete acini or tubules lined by a single layer of cuboidal or columnar cells. Both types possess the essential criteria for classification as GCC [23]. The tubular type is more closely related than goblet cell type to the usual appendiceal carcinoid in both histological and prognostic characteristics and is probably an evolutionary stage in the development of the goblet cell type [23]. These cells are located below the crypts of Lieberkühn and usually do not involve the mucosa [3]. Cells are usually uniform with pattern of infiltration being nests, rosettes, or clumps without a distinct lumen [2]. As the cells are distended with mucin, they have crescentric nuclei arranged around the periphery of the clumps, resembling goblet cells or signet-ring cells [2]. A few cells are multinucleated [23]. A high cellular proliferation rate with dysregulation of the cell cycle and minimal cytologic atypia is reported in most studies [15,16,29,19]. Some studies do mention that the mitotic rate is low [10,23].

The two types of granules (neuroendocrine and mucinous) that normally exist in GCC cells are not mixed with one another in the same cell and are completely separate [31,21] though in one type, the granules are membrane-bound and sometimes fused (confluent) with adjacent granules, constituting a loose reticular matrix [32]. Electron microscopy shows pleomorphic granules including mucinous vacuoles of varying sizes and membrane-bound neuroendocrine molecules [15,19]. Because of copious production of mucin, which is predominantly acid in type, the cells are strongly positive for mucicarmine, perodic acid-Schiff diastase, and alician blue [15,22]. This mucin is a nonsulfated acid mucopolysaccaride and resistant to sialidase digestion [23]. The degree of mucin production ranges from cells distended by coalescent mucin granules to cells with variable numbers of smaller, dark granules [33].

Because GCC is considered to be derived from crypt cells, there are other immunohistochemical techniques for their diagnosis including Cytokeratin 20 (CK20), CK7 (with the positivity of CK20 more than CK7), neuron-specific enolase (NSE), chromogranin A, serotonin, lysozyme, PGP 9.5, IgA and vimentin [7,14,24,27,33,34]. In general, because of some neuroendocrine granules in the cytoplasm, the cells are reactive for NSE, chromogranin A, Synaptophysin, Gerimelius stain, Fontana-Masson stain, serotonin, substance P, peptide YY, glucagon alpha-chain and S-100 protein [26,35]. All GCCs display extensive SC (secretory component) reactivity [35]. It is important to mention that synthesis of SC and transport of IgA are not functions of APUD cells and probably the source of this IgA is from circulating dimeric IgA synthesized by lamina propria plasma cells [36]. In one study, some tumors showed weakly positive staining with antiserum to somatostatin [27]. The presence of transitional or mixed endo/exocrine cells can be proved only in semi thin sections stained with a combination of the immunocytochemical reaction for 5-HT and alician-blue [35]. It is also important to mention that the reaction for NSE is somewhat weaker in GCC than in typical carcinoids [35]. Some eosinophilic (and phloxinophilic) granules, similar in size to basal granules of crypt base cells, i.e., Kulchitsky cells, are present in basal aspects of some cells [19].

Carcinoembryonic antigen (CEA) is one of the markers that are diffusely positive on the plasma membrane of tumor cells in GCC [7]. Höfler *et al *mentioned that SC-, lyzozyme- and CEA-reactivity was strictly limited to the exocrine tumor areas and was not found in endocrine cells of the normal mucosa [35]. In one case with bilateral ovarian metastasis, tumor cells were strongly positive for CEA and CA19-9 [37]. Tumor cells of GCC of the appendix show a preserved expression of beta-catenin and a reduced expression of E-cadherin, compared to the expression of these proteins in normal mucosa [7]. Most cells are positive for synaptophysin [15] and anti p53 antibody [38]. Sometimes expression of cell proliferation markers p53, cyclin D1 and p21 are increased while others such as p16 is down regulated. [15].

The characteristics of GCCs that somewhat simulate ordinary carcinoids are: 1) intramural position; 2) continuity with basiglandular cells of the mucous membrane; and 3) generally well-differentiated nature as well as the presence of goblet cells, argentaffin or argyrophil cells and Paneth cells in varying proportions [2]. Normally goblet cells develop independently of Paneth cells but in special circumstances, Paneth cells might represent a reserve from which goblet cells could develop [2]. Paneth cells can be seen in between one third and one half of cases [33]. Paneth cell differentiation is predominant in tumors that metastasize to the ovaries [16] and both argyrophil positive cells and Paneth cells can be seen in lymph nodes and small bowel metastases [33] but only one study mentioned that Paneth cells were not found in the metastatic deposits [23]. In addition, Paneth cells are located at the periphery of cell nests [31]. Paneth cells are not the major cells in gastrointestinal mucin-producing adenocarcinoma but they are prominent in GCC of the appendix [18]. One study mentioned that the presence of Paneth cells, like APUD cells can be as part of the neoplastic process or because of the migration [36]. Argentaffin cells are one of the cell types in the GCC cellular mass that in addition to goblet cells proliferate during tumor growth and both cell types are invasive [19]. Argyrophil cells are found in nearly 88% of GCCs and argentaffin cells in nearly 50% of GCCs [23]. The demonstration of argentaffin or argyrophil cells in a high percentage supports the classification of GCC within the spectrum of carcinoids. However, the mere presence of these cells in a tumor is not pathognomonic for carcinoid. Argentaffin and argyrophil cells are also seen in gastric and intestinal adenocarcinoma and cystadenocarcinoma of the appendix, although these cells are not as numerous as GCCs [23]. The Hamperl-Masson and diazo reactions for argentaffin granules are positive predominantly in supranuclear position in the nonvacuolated cells [21]. In addition, the argentaffin cells also react positively to the Sevier-Munger argyrophil stain [20]. The Churukian-Schenk and Grimelius staining have been used for argyrophil granules too [39,5]. In general, approximately three-fourths of the neoplastic cell nests have sharply defined cytoplasmic areas that contain highly electron-dense, pleomorphic enterochromaffin granules (EG]. Enterochromaffin cells are situated basally in the cell nest and have processes that extend either circumferentially or, less commonly, a short distance toward the center of the cell nest. Because of the processes, the number of these cells per cell nest may be overestimated when viewed by light microscopy and some nests do not contain enterochromaffin cells [31].

In summary, GCCs are derived from undifferentiated stem cells and are different from typical carcinoids that originate from endocrine cells in the mucosal stroma [35] and the degree of integration of the goblet cells versus APUD cells exists across a spectrum varying from pure GCC to pure carcinoid tumor [32]. GCC cells have two type of granules which are not mixed and are mainly acid mucinous and can be recognized by different histochemical staining.

When microscopically evaluated, atypical cells show a prominent submucosal growth pattern with diffuse infiltration from the submucosal layer to the subserosal layer forming a microlumen structure. The growth pattern of atypical cells is within the lamina propria and extends through the muscularis propria into the subsequent serosa without destroying the appendiceal structure [26]. Generally, GCC affects the base of the glands and infiltrates the lamina propria and other layers [18]. In addition, in electron microscopy, there are equal number of elongated cells containing many mitochondria and electron dense granules compressed between goblet cells. The cell membrane of both goblet cells and elongated cells shows complex interlocking of villous processes 90–100 nm in width [19]. In general, elongated cell nests are oriented parallel to the muscle that they invade and some branching can occasionally being observed. Many of these cells are invested by variable layers of fibroblasts [19]. In the mucosa, single endocrine cells can be observed incorporated in the exocrine mucus producing tissue. They are located at the periphery of tumor cell clusters, making long cell processes [35]. Höfler *et al *explained features of GCC as 1) isomorphous tumor structure, 2) the presence of adenoid tumor cell groups in the intestinal wall, 3) intact mucosa overlying the tumor, and 4) the presence of endocrine cells [35].

Macroscopically, in most cases, there is no well-defined mass. There maybe areas of indurations in the wall of the appendix or stenosis of the lumen with diffuse fibrous thickening of the appendix, which draw attention to the possibility of tumor [10,40]. The depth of bowel wall invasion is defined as follows: T1, tumor invading the submucosa; T2, tumor invading the muscularis propria; T3, tumor invading through the muscularis propria into the subserosa; and T4, tumor perforating the visceral peritoneum or directly invading other organs or structures [41]. The hallmark of GCC is the presence of individual glands separated by smooth muscle or stroma and the lining cells contain intracytoplasmic mucin [28]. This tumor is characterized by its tendency to arise within the lower lamina propria and to infiltrate extensively through the appendiceal wall without much tissue destruction [40]. Architecturally, GCC arises from the base of the glands and spares the luminal mucosa. Unless perforation has occurred, the outer muscular coats of the appendix are relatively well preserved [5].

Regarding the anatomical location of the tumor, one study mentioned that the tumor was present in 70% in the distal segment of the appendix [42]. Regarding peri-appendiceal involvement another study mentioned that more than 50% serosa or mesoappendix was involved, and in more than 60% the lymph nodes were not involved [1]. Generally, diffuse infiltration into the peri-appendiceal fat and perineural invasion is seen in most cases [15]. The tumor cells are present in the muscle layers and serosa of the appendix, in deeper layers of the appendix, the tumors are more distended, with mucin causing a ballooning effect and sometimes there are pools of extracellular mucin in the muscular coat [10]. The tumor with the most differentiated histological pattern is closest to the lumen in all cases [10]. In some cases the only gross feature is a localized yellow to grayish-white, soft or gelatinous appendiceal thickening [23]. The growth of this tumor may be retarded by fibrosis and muscle spasm that impairs the blood supply with some degeneration of tumor islands [19]. Exclusive of tumors that metastasize, the lesions for the most parts are uniform in size and shape [23].

The majority of the metastasizing GCCs extend to the serosa of the appendix though serosal involvement is not necessarily an ominous finding [23]. Metastases usually do not occur from any GCC's that does not extend beyond the muscularis propria [23]. In some cases where metastasis occurs, immunoreactivity for serotonin is seen only in the primary tumor, but not in the metastasis, and therefore it is hypothesized that that the mucinous component might have a selective ability to metastasize [43].

Review of all diagnosed patients has shown that the mesoappendix was involved in 21.64% of cases and perineural involvement as the second most common tumoral extension was seen in 2.06% of all patients.

### Clinical manifestations of GCC

Clinical diagnosis of GCC is seldom made preoperatively. Most of the patients present with signs and symptoms of an acute appendicitis due to luminal obstruction [2,6]. The tumor cells proliferate sparsely and do not form nodules. The appendiceal wall thickens diffusely with fibrous proliferation, leading to contraction of the appendiceal lumen, which is the cause of the appendicitis [26]. Other manifestations include asymptomatic patients, intussusception, a palpable mass, gastrointestinal bleeding, increasing abdominal girth, chronic intermittent lower abdominal pain, and secondary genitourinary complications [6,45]. The duration of symptoms in symptomatic patients is in the range of 12 hours to 90 days [2]. In a number of patients the tumor is identified incidentally during an operation performed for some unrelated entities [2]. In women, they may also present as Krukenberg tumors to the ovaries [46].

In those patients where the tumor metastasizes to the ovaries, half of them primarily presented as an ovarian mass [47]. Some rare instances of clinical presentations include mesenteric adenitis, intestinal obstruction (caused by tumor infiltration of the terminal ileum) and anemia (caused by extensive tumor infiltration of the ceacum with mucosal ulceration) [27]. Höfler *et al *reported one GCC with mucosal ulceration [35]. Although serotonin can be detected immunohistochemically in these tumors, carcinoid syndrome at any stage is rare [27]. Carcinoid syndrome or Cushing's-like syndrome, resulting from the systemic effect of peptide secretion, indicates liver metastasis [48]. Sometimes mucocele may be seen as a parallel pathological finding around the involved GCC of appendix [17]. A very rare complication of GCC of the appendix is appendico-vesical fistula. In this report, the patient clinically presented with urological symptoms. After laparotomy the abscess and granulation tissue involving the appendix and bladder wall was detected and GCC was confirmed by histopathological examination [22].

Review of the literature has shown that the most common clinical presentations for GCC of the appendix in order of frequency are as follows: acute appendicitis 22.5%, asymptomatic 5.4%, non-localized abdominal pain 5.15% and abdominal mass in 3.09%.

### Diagnosis of GCC

GCC is included under the general heading of mixed (composite) glandular-endocrine-cell carcinomas [49]. The diagnosis is almost always made postoperatively [3]. GCCs often present clinically as a diffusely inflamed appendix, with the diagnosis made by histological examination after surgery [15]. GCCs do not form discrete tumors and are not suspected during operation or at gross examination of the appendix. So, the surgical resection margins should be examined microscopically in all appendectomy specimens, especially in older patients, in whom these tumors most often occur [28].

GCC should be sharply distinguished from ordinary carcinoid tumors of the appendix, since they could behave biologically like carcinoma [2]. The mode of growth with concentration of the tumor elements within the basal part of the mucosal lamina propria, the pattern of invasion, growing diffusely through the layers of the wall without causing necrosis or much tissue destruction are more compatible with a carcinoid than with an adenocarcinoma [30]. GCCs can be easily diagnosed when they are of pure type [28]. The distinction between GCC and conventional carcinoid is based on a quantitative estimation of mucin production, since many carcinoids contain a little mucin [40]. In addition, in contrast to conventional carcinoids, GCCs contain few APUD cells and show positive staining of tumor cells for lysozyme, SC, and IgA [36]. For discriminating carcinoids and GCCs of the appendix from other gastrointestinal carcinomas, the predominance of signet-ring cells is found in sites often involved by metastatic signet-ring cell carcinomas of the gastrointestinal tract, whereas predominance of the carcinoid-cell type is found in sites most often involved by metastasis of conventional carcinoid tumors of appendix and GCC [43].

The resemblance of some areas in GCCs to Brunner's glands has been noted. However, the mucin in the latter is essentially neutral, whereas it is predominantly acid in GCC of the appendix [10]. In addition, in the face of excessive mucus production and an infiltrative pattern, differentiation from a mucinous adenocarcinoma remains a real challenge [17]. Histological transformation to ordinary adenocarcinoma has been observed occasionally in metastatic lesions of GCC, but most adenocarcinomatous lesions reported so far were still accompanied by varied numbers of argyrophilic tumor cells [37]. So the number of argyrophilic or argentaffin cells, as a tumor component is allegedly an indicator of the malignant potential in GCC's of the appendix with nonmetastasizing tumors commonly containing numerous neuroendocrine-type cells [37]. GCC of the appendix can be regarded as a peculiar type of adenocarcinoma, particularly when the number of neuroendocrine-type cells is limited [37]. In histological aspects for differential diagnosis, mucinous carcinomas differ from GCCs by the presence of glandular fusion and lack of glandular lumina within the mucin lakes [28]. Distinction between mucinous cystadenocarcinoma of appendix and GCC should also be considered. In the former, the mucosa lining the lumen is itself neoplastic, unlike GCC where the overlying epithelium appears normal with neoplastic transformation occurring from the deeper layers of the mucosa [17].

In most reports of metastasizing GCCs, the patients present with spread beyond the appendix at initial diagnosis [28]. In several reported cases, the ovarian masses were detected before the appendiceal tumor was recognized. In this clinical setting, the danger of misdiagnosing either a primary ovarian neoplasm or a metastasis from another site is high [5]. When mucin producing solid ovarian tumor is found without an extra-ovarian primary tumor during operation for another reason, then a diagnostic appendectomy should also be performed concomitantly [48]. When cystic mucinous tumors of the ovary showing bilateral involvement and/or stromal invasion by signet ring-type cells are encountered, the possibility of metastasis from an appendiceal GCC should be considered, irrespective of the presence or absence of argyrophilic tumor cells [37]. In-labeled octreotide scintigraphy is useful for diagnosis and staging of metastasis.

### Paraclinical aspects of GCC

The white blood cell count is generally elevated with predominantly polymorphonuclear leukocytes and a "shift to the left," especially in patients presenting with acute symptoms [2,20]. However, it can be within normal range in some patients [2]. Generally, c-reactive protein is slightly elevated [7]. In one reported case with metastasis to ovaries, serum level of CEA was elevated [37] and in another report, plasma serotonin level was elevated preoperatively but these do not occur frequently [5]. Urinary 5-hydroxyindoleacetic acid (5-HIAA) level is estimated to be mostly within normal limits [27]. In cases with liver metastasis, liver enzymes and serum bilirubin will be elevated [43]. One of the rare complications of GCC of the appendix and all mucinous tumors of the appendix and or ovary is the occurrence of pseudomyxoma peritonei. This is a neoplastic disease of MUC2-expressing goblet cells, which collectively overexpress MUC2 because of their increase in number. Goblet cells of the appendix express both MUC2 and MUC5AC mucins but primary ovarian mucinous tumors only express MUC5AC. So measuring MUC2 is a specific marker for diagnosis of appendiceal induced pseudomyxoma peritonei [49,51].

X-ray of the abdomen shows dilated small bowel loops with fluid levels when obstruction occurs [45]. Cross-sectional imaging with CT is the technique of choice for any suspected appendiceal mass to rule out a tumor. In this technique the findings for GCC reflects the infiltrative nature of the tumor, with mild but diffuse mural thickening [6].

### Surgical therapy of GCC

The site of the tumor within the appendix is not important for the choice of treatment [51]. In general, therapy is surgical. Even though GCC has a more aggressive phenotype than benign carcinoid tumors, the prognosis is generally good in a patient who is treated by simple appendectomy. Appendectomy remains the treatment of choice in the majority of patients. In some patients however a more radical procedure is indicated especially in the presence of diffuse appendiceal involvement [38,2,3]. Recently Varisco *et al *emphasized that in patients with no concomitant caecal involvement and low-grade tumor's histology; a simple appendectomy is enough [52].

Some studies recommend that because GCCs are among the semi high-grade malignant carcinoids with unpredictable behavior, in addition to simple appendectomy a right hemicolectomy should also be performed [45,42]. The general consensus remains that a right hemicolectomy should be performed when one or more of the following criteria are noted: 1), cellular undifferentiation; 2), increased mitotic activity; 3), involvement of the base of the appendix with caecal wall infiltration; 4), lymph node metastasis; and 5), tumor size greater than 2 cm. Invasion only into the mesoappendix or histological findings of intralymphatic or perineural invasion does not necessarily, by itself, require a more radical procedure [2]. However, if it cannot be confirmed that the base of the appendix is tumor-free, a right hemicolectomy is indicated due to the frequency of regional nodal metastases [27]. One study explained the indication of right hemicolectomy based on: incomplete excision of the tumor, foci of dedifferentiation and two or more mitotic figures per ten high power fields [25].

In addition to right hemicolectomy, some authors also advocate bilateral oophorectomy in female patients because of the possibility of ovarian metastasis [46,48]. Conversely, due to the tendency of GCCs affinity of spread to the ovaries, it is recommended that an appendectomy be routinely performed in patients with Krukenberg's tumor of the ovary where no gross obvious primary neoplasm can be found [53]. So, if an intraoperative histological evaluation reveals metastatic ovarian tumor, and at the same time, an obvious primary lesion is apparently absent, thorough exploration of the appendix is mandatory and routine appendectomy is recommended even if the appendix appears macroscopically normal [46]. In disseminated disease of the appendix in the abdomen, radical surgery is not recommended because it is doubtful that the course of the disease will be altered [48]. Thus, until better prognostic markers can be identified, an elective right hemicolectomy offers the best chance of controlling the disease and preventing recurrence in such patients [54,42]. As the route of metastatic spread of GCC's is through lymphatic and venous drainage, one should check the areas on the anterior surface of the third portion of the duodenum and above the origin of the ileocolic artery for metastatic deposits [55].

Even though the GCC of the appendix is not a complete high-grade malignant tumor, it can recur and metastasize so complete removal is imperative [10]. It is emphasized that recognition of this disease during the appendectomy from clues such as an abnormally thickened appendiceal wall, or the retention of mucinous material in the lumen should be encouraged [7].

Review of all reported GCCs showed that simple appendectomy was performed in nearly 24.57% of cases especially in the early reports. The most common surgical treatment of choice was appendectomy with right hemicolectomy (RHC) especially in the recent reports (34.7%). Oophorectomy was also performed in nearly 11.39% of female patients. In conclusion, because of the moderately aggressive behavior of the tumor, it is recommended that in all patients in addition to appendectomy a right hemicolectomy is also performed. As the mean age of the patients is over 50 and ovaries are the most common sites of metastasis, and bilateral involvement of the ovaries is the commonest metastatic behavior, bilateral salpingo-oophorectomy with total abdominal hysterectomy (TAH-BSO) is also advocated in selected female patients. Additional file: 1 summarizes the management of all diagnosed GCCs based on chronological reporting.

### Adjuvant therapy of GCC

Peritoneal carcinomatosis from GCC origin is as invasive as peritoneal surface malignancy from colorectal adenocarcinoma. Patients in whom complete or near-complete surgical removal is possible should be considered for cytoreduction in combination with intraperitoneal chemotherapy [56].

As GCC's have this capacity to seed diffusely into the peritoneal cavity, innovative chemotherapy regimens similar to gastric cancer chemotherapy is advocated though in one study chemotherapy was administered without successful response [3]. One study of GCC with subserosal involvement advocated postoperative chemotherapy with 5-FU, Leucovorin, and CPT-11 [8]. Some authors mention that even small tumors early in the course of the disease will cause appendix obstruction and perforation which results in release of tumor cells into the free peritoneal cavity and this almost occurs before lymph node metastasis and therefore advocate the routine usage of interaperitoneal chemotherapy. There is no added benefit using either combination of mitomycin C plus 5-FU versus cisplatin plus Adriamycin [57]. There is also one report of a patient with GCC of the appendix and bilateral ovarian metastasis receiving X-ray radiation therapy as an adjuvant and after 8 months follow-up, no recurrence was detected [55]. Wojcik *et al *reported the first case of GCC on peritoneal fluid cytology in a woman with a prior history of carcinoid tumor of the appendix and the patient underwent 11 courses of Adriamycin and alpha-interferon with unknown documented follow-up [49]. A regimen of 5-FU and streptozocin was recommended in a case with appendiceal GCC and bilateral Krukenberg's of the ovaries and the patient was alive after 7 months follow-up [53]. There is also another report of GCC with Krukenberg's tumor, which advocated that cisplatin-based chemotherapy has some therapeutic benefits [46]. In general, Krukenberg's tumors subgroup due to appendiceal GCC has a survival advantage in patients receiving surgery and adjuvant therapy compared to surgery alone [34]. In one reported case with bilateral ovarian metastases patient was treated with carboplatin, cyclophosphamide and pirarubicin and followed by 9 months outpatient tegafur uracil (UTF) [37]. A recent study mentioned that a folfox chemotherapy that includes oxaliplatin combined with 5-FU and leucovorin is helpful in complete and persistent remission of a GCC case with metastasis to ovaries, peritoneal cavity and lymph nodes [58]. A summary of all reported GCCs and their management including chemotherapy is shown in Additional file: 1.

Even though it was not prescribed routinely, most protocols include 5-FU. As peritoneal seeding can occur before lymph node metastasis and there is proven advantage of chemotherapy in metastatic patients, 5-FU is therefore highly recommended in all GCC patients. Radiotherapy was only ordered in 4 cases without any specific consequence.

### Follow-up of patients with GCC

Metastasis occurs in nearly 20% of the cases [16]. In general, metastatic ability is calculated in a range from 0 to 50 % [40]. The most common route of metastasis is through lymphatic vessels, trans-celomic and intraperitoneal invasion [27,33]. However, hematogenous metastasis to the liver or other distant organs is rarely mentioned [33]. Metastasis beyond the appendix is more frequent in older patients [27]. Metastasis from GCCs of the appendix has been described to the ovaries, pelvis, abdominal cavity, rib, vertebra, and lymph nodes [2,52]. The ovary is the most common target site of metastasis of GCC and metastatic lesions sometimes show a histological picture of mucin-producing adenocarcinoma [37]. In general, when metastatic spread occurs, it shows a predilection for the peritoneum and ovaries, with peritoneal carcinoidosis [43]. GCC's have a propensity to metastasize to the ovaries indistinguishable from the typical Krukenberg's tumor [46]. Primary ovarian carcinoids are invariably unilateral, whereas metastatic carcinoids to the ovaries are almost always bilateral or unilateral with extraovarian involvement [34]. In female patients with metastatic GCCs, ovarian involvement is seen in almost 90% of cases and is the source for further tumor dissemination [16]. Liver metastasis with abnormal liver function tests and hepatomegaly was also seen occasionally [27]. In metastatic deposits to organs including ovaries, fallopian tubes, uterus, and colon the histology is different and mostly composed by signet-ring cells without typical carcinoid cells but the metastases to liver and lymph nodes show a pattern of typical midgut carcinoids with signet-ring cells centrally and carcinoid cell type peripherally [43]. Also in rare cases only the mucinous component may spread and metastasize [39].

In-labeled octreotide scintigraphy is the most sensitive imaging modality in the diagnosis and staging of metastatic disease. Plasma chromogranin A is the most important blood marker available with levels being raised in 80–100 percent of patients with neuroendocrine tumors including GCC. Chromogranin A levels correspond to tumor load and levels above 5000 μg/l predict a poor outcome. Therefore, all patients with GCCs warrant investigation with plasma chromogranin A concentration, 24-h urinary levels of 5-HIAA, CT and In-labeled octreotide scintigraphy [45]. Where appendectomy has been done as the only treatment, prolonged follow-up is recommended and may involve laparoscopy in view of the risk of trans-celomic spread [27]. One study mentioned that pancreatic polypeptide might be useful as a tumor marker for follow-up purposes [27].

Review of the literature has shown that 11.2% of the patients were diagnosed with concomitant distant metastasis with ovaries being the most common site (3.60%) followed by abdominal carcinomatosis (1.03%). In addition, local lymph node involvement was seen in 8.7% of patients at the time of diagnosis. After surgical treatment, metastasis occurred in 4.1% of patients most commonly with abdominal carcinomatosis in 1.37% followed by ovaries in 0.85%. In conclusion, when GCC is suspected, both the ovaries and the peritoneum should be examined both during surgical extirpation and during follow up.

### Prognosis of GCC

The prognosis of these tumors is estimated to be somewhere between carcinoids and well-differentiated adenocarcinomas of the appendix [3]. Prognosis is mostly favorable except in those patients who present with the more virulent form, delayed local recurrence, lung metastases or dual neoplasia e.g., GCC/epithelial neoplasia. In dual neoplasia prognosis depends on the stage and grade of the other component [3]. The association of GCC of the appendix with other cancers also clearly worsens the prognosis of the patient [2]. The associated carcinomas are most commonly carcinoma of the urinary bladder, prostate, ovaries, stomach and breast [2]. Other study however suggested that the prognosis of dual appendiceal GCC/epithelial neoplasia would be no worse than for either of the components alone [12]. There are also two reported rare cases of GCC and mucinous cystadenoma of the appendix with no information on their prognosis and outcome [29]. Involvement of the base of the appendix with only limited caecal wall involvement may still carry a favorable prognosis. Extension of the tumor beyond the appendix to the ovaries or the abdominal cavity, however, greatly worsens the prognosis [2]. In general, when tumor metastasis to the ovary is present, the prognosis is poor with a mean survival of 7 to 9 months [47]. Widespread intraabdominal dissemination can be fatal even in a short time [48].

Histological features such as nuclear pleomorphism and mitotic activity show no significant correlation with staging or prognosis [27]. On the other hand, some authors say the vast majority of GCCs that metastasize have spread beyond the serosa, suggesting that stage is another important prognostic factor [16]. Tumor characteristics that predict aggressive behavior include size, histological subtype and mesoappendiceal involvement [45]. Discussion regarding tumor size is controversial. Some studies say that size is not a good indicator for prognosis because these lesions do not always form discrete masses [3]. Other studies say the tumor size is only related to the metastatic potential and tumors larger than 2 cm are associated with a poorer prognosis. However, even smaller tumors do occasionally metastasize [48]. Angioinvasion should be regarded as a feature of more malignant behavior. In general, serosal involvement and mesoappendiceal extension are more predictive of outcome than lymph node status [45]. One study reported that those GCC tumors that show positive staining for pancreatic polypeptide behave in an aggressive fashion [27].

As a rule, survival for GCC of appendix is not significantly worse than that for patients with malignant carcinoid when adjusted for age and extent of disease at presentation, and is similar to survival after treatment of a low-risk adenocarcinoma [45]. One report referred to increasing age and evidence of tumor spread as poor prognostic factors [27]. In addition, reduced E-cadherin staining and overexpression of p21 need further assessment as future prognostic follow-up markers [59]. Another study mentioned that 20% of appendiceal GCCs that metastasize result in death [16]. The reported 5-year survival of GCC is between 60% and 84% [61,15,12,60,8,53] and 16% of all cases have recurrence of the disease [54].

Generally, the behavior of GCC is unpredictable and cannot be anticipated from the tumor's diameter, as in the case with carcinoid tumors [25]. Average survival time is 4.6 years with a range of 6 months to 20 years [25].

## Conclusion

GCC of the appendix is a rare neoplasm that has varied clinical presentations ranging from an asymptomatic patient that is recognized during investigations for another problem, to acute appendicitis, the most common presentation, and toward the other end of the spectrum the metastatic presentation like Krukenberg's tumor or widespread peritoneal metastatic disease. So with this wide range of presentation, GCC should be always considered as a differential diagnosis in patients with "suspected appendicitis" with or without a mass leading to abdominal surgery. After the tumor is histologically confirmed, some factors need to be evaluated histologically as predictive factors for the biological behavior, e.g., the increased number of Paneth cells, increased mucin secretion and positivity for pancreatic polypeptide show a more aggressive and metastatic behavior and such cases should be considered for long-term follow-up. As nearly 20% show metastatic behavior and high possibility of intraperitoneal seeding before lymph node involvement, appendectomy with completion right hemicolectomy is recommended for all patients with caecal involvement and or high-grade tumors. Moreover, as the most common sites of metastasis are ovaries, in female patients, regardless of age and involvement, bilateral salpingo-oophorectomy is also recommended. In the presence of metastasis, combination chemotherapy regimes include 5-FU and leucovorin. It should be kept in mind that despite different terminology the biological difference between adenocarcinoid and carcinoma is not significant. Peritoneal carcinomatosis from adenocarcinoid of appendiceal origin is as invasive as peritoneal surface malignancy from colorectal adenocarcinoma. Patients in whom complete or near-complete surgical removal is possible should be considered for cytoreduction in combination with intraperitoneal chemotherapy. Based on the tumor histological predictors, long-term follow-up should be routinely advocated in all cases for possible metastasis. In this context, In-labeled octreotide scintigraphy is recommended for diagnosis and long-term follow-up of this neoplasm.

## Competing interests

The author(s) declare that they have no competing interests.

## Authors' contributions

**PSP: **Is the first and the corresponding author, Conception and design, Literature review with references, Drafting the article and revising, provided statistical analysis of the reviewed data, preparation of table.

**RK: **helping in the preparation and drafting the manuscript, literature update, supervision, proof-reading and revising the manuscript

## Supplementary Material

Additional file 1 Click here for file
