# Supplementary material for: Goblet cell carcinoid of the appendix
Source: World J Surg Oncol. 2005 Jun 20;3:36. doi: 10.1186/1477-7819-3-36 (PMC1182398; doi:10.1186/1477-7819-3-36)
Supplement: Additional file 1 [file 1477-7819-3-36-S1.doc]

Additional file 1. Reported cases of GCC of the appendix and their management

| **Source, year** | **Cases** | **Age**  **(range)** | **Gender**  **(M:F)** | **clinical presentation** | **Tumor extension** | **Lymph node**  **involvement** | **Distant metastasis** | **Operative procedure** | **Adjuvant**  **therapy** | **Post -management**  **complications** | **Post-management metastasis** | **follow up** | **Survival** |
| --- | --- | --- | --- | --- | --- | --- | --- | --- | --- | --- | --- | --- | --- |
| Subbuswamy et al (1974) [10] | 12 | 52.5  (37-69) | 7:5 | Acute appendicitis: 6  Chronic appendicitis:1  Abdominal pain: 4  symptomless:1 | mesoappendix involvement in 6 | unknown | None | 8 cases appendectomy, 3 cases appendectomy + RHC, 1 case appendectomy + hysterectomy | None | None | 1(ovaries) | 1 - 1 yr  4 - 2 yr  1 - 3 yr  1 - 4 yr  2 - 5 yr  1 - 6yr,  1-NA  1> 10 yr | 1yr:91.6%  3yr: 50%  5yr:16% |
| Klein (1974) [20] | 3 | 61  (45-81) | 2:1 | Acute appendicitis: 2,  Abdominal pain: 1 | None | None | None | 2 cases appendectomy + RHC , 1 case appendectomy | None | None | None | 1 4.5 yr  1-3 yr  1-9 mon | 1yr:66.6%  3yr:33.3% |
| Abt et al. (1975) [21] | 1 | 29 | 1:0 | Acute appendicitis | None | None | None | appendectomy | None | None | None | NA | NA |
| DiPaola et al. (1976) [22] | 1 | 75 | 1:0 | Dysuria & urinary incontinency | Bladder | NA | NA | appendectomy + bladder diverticulectomy + transvesical prostatectomy | None | NA | NA | NA | NA |
| Haqqani et al.(1977) [18] | 6 | 47.1  (23-60) | 2:4 | Acute appendicitis:2  PID:1  Hematuria:1  Abdominal pain:2 | None | NA | 1 (ovaries) | 4 cases appendectomy, 2 cases appendectomy + TAH-BSO | None | None | 1(suspicious) | 3-less than 1 yr  1-2yr  1-3yr  1-9yr | 1yr:50  3yr:16.6%  5yr:16.6%: |
| Warkel et al.(1978) [23] | 39 (1 of these cases was reported by Klein in 1974) | 54.1  (34-75) | 25:14 | Acute appendicitis:23  Mass:2  Bowel obstruction:1  symptomless:9  Autopsy finding:2  NA:2 | 2 (1: Omentum, 1:cecum and peritoneum) | 1 | 6(4:intra abdominal, 1: ovaries, 1:carcinomatosis) | 13 cases appendectomy, 12 cases appendectomy + RHC, 14 cases ? | None | None | 6(4:intra abdominal, 1: ovaries, 1:carcinomatosis) | 32 available  (1-7 yr) | 5yr:79.8% |
| Cooper et al.(1978)[31] | 2 | 58  (51-65) | 2:0 | Acute appendicitis: 2 | None | None | None | 1 case appendectomy, 1 case appendectomy + ileocecal resection | None | None | None | NA | NA |
| Chen et al.  (1979)[17] | 5 | 48.6  (31-61) | 3:2 | Acute appendicitis:4  Mass: 1 | None | None | None | 2 cases appendectomy, 2 cases appendectomy + RHC, 1 case appendectomy + right ovarian resection | None | None | None | 1- 12yr  1-1yr  1-4yr  1-7yr  1-3yr | 1yr:80%  3yr:60%  5yr:40% |
| Warner et al.  (1979)[19] | 1 | 62 | 1:0 | Acute appendicitis | None | None | None | appendectomy + cecal resection | None | None | None | 3yr | 1yr:100% |
| Ratzenhofer et al.  (1980)[4] | 2 | 59  (48-70) | 1:1 | NA | NA | NA | NA | NA | NA | NA | NA | NA | NA |
| Zirkin et al. (1980) [63] | 1 | 62 | 0:1 | Mass | None | Unknown | Ovaries,  Mesentery  Omentum | appndectomy, omentectomy, TAH-BSO | Unknown | NA | NA | NA | NA |
| Olsson et al.  (1980)[30] | 4 | 35  (20-54) | 0:4 | Acute appendicitis:2,  Abdominal pain:2 | None | None | None | 1 case appendectomy + TAH-BSO, 1 case appendectomy + ileocecal resection + TAH-BSO + ileotransversotomy, 2 cases appendectomy | 1 case cytostaticum | NA | 1(carcinomatosis) | 1-1yr  1-2yr  2-3yr | 1yr:75% |
| Isaacson  (1981)[36] | 4 | NA | NA | NA | NA | NA | NA | NA | NA | NA | NA | NA | NA |
| Rodriguez et al  (1982)[32] | 1 | 49 | 1:0 | Abdominal pain | 1(perineural invasion) | None | None | appendectomy | None | NA | NA | NA | NA |
| Heisterberg et al.  (1982)[55] | 2 | 58  (57-59) | 0:2 | Abdominal pain:2 | None | 1 | 2(Ovaries) | 1 case appendectomy + omentectomy + TAH-BSO, 1 case appendectomy + RHC + TAH-BSO | 1 case adriamycin + 5-FU, 1 case X-ray radiation therapy | None | None | 2-1yr | 1yr:100% |
| Edmonds et al.  (1984)[5] | 10 | 44  (23-65) | 5:5 | Acute appendicitis:5  Abdominal mass:3  Ovarian mass:2 | NA | NA | None | 5 cases appendectomy, 3 cases appendectomy + RHC, 1 case appendectomy + RHC + oophorectomy, 1 case appendectomy + RHC + TAH-BSO | 2 cases chemotherapy (agent = ?)  1 case radiotherapy | NA | 2(Ovarian metastasis)  1(abdominal carcinomatosis)  1(lymph node) | 4--1yr  2-3yr  1-5yr | 1yr:60%  3yr:20%  5yr:10%- |
| Höfler et al. (1984)[35] | 5 | 65  (55-67) | 4:1 | NA | 1(Mesenteri lumen) | 1 | 1(Lymph node) | 4 cases appendectomy, 1 case appendectomy + ileocecal resection | NA | NA | NA | NA | NA |
| Hirschfield et al. (1985)[53] | 1 | 33 | 0:1 | Abdominal pain | Mesoappendix | None | Ovaries | TAH-BSO, omentectomy, appendectomy | 5-FU, streptozocin | NA | NA | 1yr | 1yr:100% |
| Bak, Jorgensen (1986)[43] | 1 | 74 | 0:1 | Abdominal pain | Mesoappendix and  Perinural | 1 | Liver | Because of liver metastasis, no surgery was performed and the patient died two weeks after admission | NA | NA | NA | 1yr | 1yr:100% |
| Watson et al. (1987) [33] | 6 | 62.1  (39-76) | 5:1 | NA | NA | 4 | 1(liver) | appendectomy + RHC | NA | NA | NA | 2-2yr | 1yr:100%  2yr:16% |
| Bak, Asschenfeldt (1987) [40] | 20 (1 case was previously reported by Bak, Jorgensen in 1986) | 58.8  (29-84) | 6:14 | NA | 8 (Perineural invasion)  6 (Vascular invasion) | 6 | 1(Pancreatic metastasis)  1(Peritoneal carcinoids)  1(Liver)  1(Plural effusion) | 1 case no operaration, 9 cases appendectomy, 5 cases appendectomy + ileocecal resection, , 4 cases appendectomy + RHC, 1 case appendectomy + TAH-BSO + omentectomy | 1 case raditherapy + chemotherapy (agent = ?) | NA | None | mean:  5yr 9mon | 1yr:55%  3yr:30%  5yr:20% |
| Miller et al. (1988)[39] | 1 | 49 | 0:1 | Abdominal mass | Perineural invasion  wall invasion up to the ileocecal valve | 1 | None | TAH-BSO, appendectomy, RHC | cytoxan, adriamycin, cisplatin, 5-FU | None | None | 2yr | 1yr:100% |
| Berardi et al. (1988)[2] | 76 | 53  (20-81) | 45:31 | Acute appenditis:49  Abdominal pain:11  Symptomless:  15 | NA | NA | NA | 55 cases appendectomy, 17 cases appendectomy + RHC, 3 cases appendectomy + RHC + TAH-BSO, 1 case ? | None | NA | 2:Yes  (location:un-known) | 21:5yr | 5yr:  32.89% |
| Burke et al. (1989)[28] | 33 (16 cases of these series was previously reported by Warkel) | 53  (31-71) | 19:14 | Acute appendicitis: 25  small bowel obstruction:1  Abdominal mass:1  Symptomless:6 | None | None | None | 20 cases appendectomy, 13 cases appendectomy + RHC | None | 1 (Abcsess formation) | None | mean:19 mon | 1yr:75% |
| Park et al. (1990)[42] | 10 | 56  (24-80) | 8:2 | Acute appendicitis:6  Abdominal mass:3  Abdominal pain:1 | None | 6 | None | 6 cases appendectomy, 4 cases appendectomy + RHC | None | NA | 2 yes  (location:un-known) | 8-2yr | 5yr:60% |
| Ikeda et al. (1991)[37] | 1 | 42 | 0:1 | Acute appendicitis | None | None | None | appendectomy + TAH-BSO + omentectomy | carboplatin (paraplatin) + cyclophosphamide + pirarubicin and for outpatient under tegafur.uracil (UFT) | NA | ovaries | 3yr | 1yr:100% |
| Anderson et al. (1991)[27] | 11 | 58  (24-76) | 3:8 | Acute appendicitis:5  Abdominal mass:3  Mesenteric adenitis: 1  Intestinal obstruction:1  Anemia: 1 | 3 (Cecum infilteration)  7:Mesoappendix | 2 | None | 5 cases appendectomy, 6 cases appendectomy + RHC | None | NA | 1: Liver  1:Peritoneal seeding | mean:32 mon | 1yr:100%  3yr:72% |
| Gallegos et al. (1992)[25] | 1 | 59 | 1:0 | Intestinal obstruction | None | None | None | appendectomy + RHC | None | None | None | NA | 5yr:73.2% |
| Zea-Iriarte et al. (1994)[14] | 1 | 47 | 1:0 | Acute appendicitis | Perineural invasion  Vascular invasion | None | None | appendectomy | None | None | None | NA | NA |
| Butler et al. (1994)[3] | 9 | 58  (31-73) | 7:2 | Acute appendicitis:4  Abdominal pain:4  Symptomless:1 | None | NA | 4(ovaries) | 3 cases appendectomy, 1 case appendectomy + RHC, 5 cases appendectomy + RHC + TAH-BSO + omentectomy | 2 cases chemotherapy (agent = ?, but similar to gastric cancer) | NA | Peritoneal metastasis | 3-<1yr  1->1yr  3-2yr  1->2yr  1-11yr | 1yr:66.6% |
| Al-Talib et al. (1995)[29] | 2 | 59  (54-64) | 0:2 | Acute appendicitis:1  Abdominal pain:1 | None | None | None | appendectomy | NA | NA | NA | NA | NA |
| Argani et al. (1995)[62] | 1 | 70 | 0:1 | Acute appendicitis | None | None | Noner | appendectomy + RHC | None | NA | Liver | 7yr | 1yr:100% |
| Carr et al.  (1995)[12] | 4 | 49  (22-92) | NA | Acute appendicitis:  (?)  Abdominal pain:(?) | NA | NA | NA | NA | NA | NA | NA | mean:  57 mon | NA |
| Klein EA et al. (1996)[34] | 1 | 66 | 0:1 | Abdominal pain,vaginal spotting | Mesoappendix | 1 | Ovaries | TAH-BSO + appendectomy + RHC | cisplatin, leucovorin, 5-FU,  radiotherapy | Myocardial infarction | Abdominal carcinomatosis | Died  after 20 mon | 1yr:100% |
| Ramnani et al. (1999)[16] | 22 | NA | NA | NA | Adjacent fat :2 | NA | 1(ovary, peritoneum, urinary bladder) | NA | NA | NA | NA | NA | NA |
| Tjalma et al.(2000) [48] | 1 | 43 | 0:1 | Abdominal mass | Adjacent fat,  Mesoappendix | None | Ovaries,  Omentum,  Peritoneum,  Diaphragm | Omentectomy, BSO, appendectomy, RHC | None | None | NA | Died after 19 mon | Iyr:100% |
| Kanthan et al. (2001)[15] | 7 | 59.7  (34-82) | 5:2 | Acute appendicitis:6  Intestinal obstruction:1 | Perineural invasion:7 | NA | None | appndectomy + RHC | None | NA | 1:Lung | mean:  18 mon | 1yr:100% |
| Kuroda et al. (2001)[7] | 1 | 58 | 1:0 | Acute appendicitis | Perineural invasion | None | None | appendectomy | None | None | None | 1 mon | NA |
| Mandai et al. (2001)[46] | 1 | 35 | 0:1 | Abdominal mass | None | None | Ovaries | appendectomy, omentectomy, TAH-BSO | cisplatin, adriamycin, cyclophosphamide, 5-FU | Intestinal obstruction | Abdominal carcinomatosis | Died after 24 mon | 1yr:100% |
| Grain et al. (2002)[58] | 1 | 58 | 0:1 | Abdominal pain | None | Multiple retroperiton-eal lymph nodes (10) | Ovaries, peritonum | appendectomy + TAH-BSO | 5-FU + oxaliplatin + leucovorin | None | None | 3 yr | 1yr:100% |
| McCusker et al. (2002)[1] | 227 | 52  (18-89) | 117:110 | NA | Mesoappendix invasion: 108 | 24 | 31(location:NA) | 119 cases appendectomy, 90 cases appendectomy + RHC, 4 case other surgery, 14 cases unknown | NA | NA | NA | NA | 1yr:97%  3yr::86%  5yr:75% |
| Li et al. (2002)[13] | 11 | NA | NA | NA | NA | NA | NA | NA | NA | NA | NA | NA | NA |
| O'Connell et al. (2002)[50] | 15 | NA | NA | NA | NA | NA | 6(Ovaries) | NA | NA | NA | Pseudomyxoma peritonei | NA | NA |
| Kende et al. (2003)[24] | 9+6 mixed GCC & AC (total 15) | NA | NA | NA | NA | NA | NA | NA | NA | NA | NA | NA | NA |
| Aizawa et al. (2003)[26] | 2 | 69.5  (60-79) | 1:1 | Acute appendicitis:2 | None | None | None | appendectomy, ileocecal resection | None | None | None | 1-1yr  1-18 mon | 1yr:100% |
| Pickhardt et al. (2003)[6] | 2 | 38.8  (38-39) | 1:1 | Acute appendicitis:1  Abdominal mass:1 | NA | NA | 1(Ovaries) | NA | NA | NA | NA | NA | NA |
| Stancu et al. (2003)[8] | 16 | 47.3  (23-74) | 8:8 | NA | Mesoappendix | 1 | NA | 10 cases appendectomy, 5 cases appendectomy + RHC, 1 case appendectomy + TAH-BSO | 1 case 5-FU + leucovorin + CPT-11 | NA | NA | mean  20 mon | NA |

Legends :

RHC : Right Hemi Colectomy

PID: Pelvic Inflammatory Disease

TAH-BSO: Total Abdominal Hysterectomy- Bilateral Salpingo-Oophorectomy
